# Evaluation of the pharmacokinetics, dosimetry, and therapeutic efficacy for the α-particle-emitting transarterial radioembolization (αTARE) agent [^225^Ac]Ac-DOTA-TDA-Lipiodol^®^ against hepatic tumors

**DOI:** 10.1186/s41181-023-00205-3

**Published:** 2023-08-14

**Authors:** Anders Josefsson, Angel G. Cortez, Harikrishnan Rajkumar, Joseph D. Latoche, Ambika P. Jaswal, Kathryn E. Day, Mohammadreza Zarisfi, Lora H. Rigatti, Ziyu Huang, Jessie R. Nedrow

**Affiliations:** 1 Department of Radiology, University of Pittsburgh School of Medicine, 5117 Centre Avenue, Suite G. 17B, Pittsburgh, PA USA; 2 Hillman Cancer Center, University of Pittsburgh School of Medicine, Pittsburgh, PA USA; 3 Department of Neurological Surgery, University of Pittsburgh School of Medicine, Pittsburgh, PA USA; 4 Division of Laboratory Animal Resources, University of Pittsburgh School of Medicine, Pittsburgh, PA USA

**Keywords:** Hepatocellular carcinoma, Lipiodol^®^, Targeted alpha therapy (TAT), Alpha-particle, Dosimetry, Actinium-225, Francium-221, Bismuth-213

## Abstract

**Background:**

The liver is a common site for metastatic disease for a variety of cancers, including colorectal cancer. Both primary and secondary liver tumors are supplied through the hepatic artery while the healthy liver is supplied by the portal vein. Transarterial radioembolization (TARE) using yttrium-90 glass or resin microspheres have shown promising results with reduced side-effects but have similar survival benefits as chemoembolization in patients with hepatocellular carcinoma (HCC). This highlights the need for new novel agents against HCC. Targeted alpha therapy (TAT) is highly potent treatment due to the short range (sparing adjacent normal tissue), and densely ionizing track (high linear energy transfer) of the emitted α-particles. The incorporation of α-particle-emitting radioisotopes into treatment of HCC has been extremely limited, with our recent publication pioneering the field of α-particle-emitting TARE (αTARE). This study focuses on an in-depth evaluation of the αTARE-agent [^225^Ac]Ac-DOTA-TDA-Lipiodol^®^ as an effective therapeutic agent against HCC regarding pharmacokinetics, dosimetry, stability, and therapeutic efficacy.

**Results:**

[^225^Ac]Ac-DOTA-TDA was shown to be a highly stable with bench-top stability at ≥ 95% radiochemical purity (RCP) over a 3-day period and serum stability was ≥ 90% RCP over 5-days. The pharmacokinetic data showed retention in the tumor of [^225^Ac]Ac-DOTA-TDA-Lipiodol^®^ and clearance through the normal organs. In addition, the tumor and liver acted as suppliers of the free daughters, which accumulated in the kidneys supplied via the blood. The dose limiting organ was the liver, and the estimated maximum tolerable activity based on the rodents whole-body weight: 728–3641 Bq/g (male rat), 396–1982 Bq/g (male mouse), and 453–2263 Bq/g (female mouse), depending on an RBE-value (range 1–5). Furthermore, [^225^Ac]Ac-DOTA-TDA-Lipiodol^®^ showed significant improvement in survival for both the male and female mice (median survival 47-days) compared with controls (26-days untreated, and 33–35-days Lipiodol^®^ alone).

**Conclusions:**

This study shows that [^225^Ac]Ac-DOTA-TDA-Lipiodol^®^ is a stable compound allowing for centralized manufacturing and distribution world-wide. Furthermore, the result of this study support the continue development of evaluation of the αTARE-agent [^225^Ac]Ac-DOTA-TDA-Lipiodol^®^ as a potential treatment option for treating hepatic tumors.

**Supplementary Information:**

The online version contains supplementary material available at 10.1186/s41181-023-00205-3.

## Introduction

The liver is a common site for metastatic disease for a variety of cancers, including colorectal cancer (CRC). Approximately 25–30% of patients diagnosed with CRC will develop metastatic disease to the liver (Engstrand et al. 2018). The American Cancer Society states the 5-year survival rate for CRC patients with distant disease to be 14% as compared to 91% for localized disease and 72% for regional disease. This discrepancy in the 5-year survival rate (2011–2017) for distant disease is influenced by the limited treatments for patients with secondary tumors in the liver.

Both primary and secondary liver tumors are fed through the hepatic artery while the healthy liver is supplied by the portal vein. Therapies, such as transarterial embolization (TAE), chemoembolization (TACE) and radioembolization (TARE), exploit these arterial routes to direct localized therapies to hepatic tumors. Current TARE-agents utilize β-particle-emitting radioisotopes, such as yttrium-90 or rhenium-188. Yttrium-90 TARE-agents utilize glass or resin microspheres and are the most advanced β-particle-emitting TARE-agents (βTARE). βTARE-agents have shown promising results with reduced side-effects but have similar survival benefits in patients with hepatocellular carcinoma as compared to TACE, the standard of care (Liapi et al. 2007; Lee et al. 2010). Similarly, in a retrospective study comparing βTARE and drug-eluting bead TACE (DEB-TACE) in patients with CRC liver metastases there was not a significant improvement in overall survival (OS) between these treatments; however, the βTARE was better tolerated (Mokkarala et al. 2019). More importantly, it has demonstrated in the DOSISPHERE trail (NCT02582034) that personalized dosimetry for βTARE significant improves the OS, highlighting the importance of dosimetry in developing TARE agents. The development of novel highly potent TARE therapies may have the potential to significantly improve the overall survival in patients with primary and secondary liver tumors.

In the past decade, the development and utilization of ɑ-particle-emitting radioisotopes has greatly expanded the field of targeted radiotherapy (TRT) (Sgouros et al. 2020; Pallares and Abergel 2022). Targeted alpha therapy (TAT) is highly potent treatment due to the densely ionizing track and a short path-length (40–90 μm) of the emitted ɑ-particles, resulting in a high linear energy transfer (LET) (50–230 keV/μm) (Sgouros et al. 2010). Only a few α-particle tracks are needed compared to thousands of β-particle tracks through a cell to cause irreparable DNA damage and cell death (Sgouros et al. 2018a; Thiele and Wilson 2018). In addition, the short path-length of the emitted ɑ-particles spares adjacent normal tissue and has shown to be efficient against chemo- and radioresistant cancer cells (Haro et al. 2012a, b). The incorporation of α-particle-emitting radioisotopes into TARE has been extremely limited, with a recent publication pioneering the field of α-particle-emitting TARE (αTARE) (Du et al. 2022). This recent study focused on the development and preliminary evaluation of [^225^Ac]Ac-DOTA-TDA-Lipiodol^®^ to serve as an αTARE-agent for treatment of hepatocellular carcinoma (HCC). This study is focused on a more in-depth evaluation of the αTARE-agent [^225^Ac]Ac-DOTA-TDA-Lipiodol^®^. The pharmacokinetics, and dosimetry of [^225^Ac]Ac-DOTA-TDA-Lipiodol^®^ and its free unbound in vivo generated decay daughters were evaluated in rodent models, including a non-tumor bearing rat intraarterial model and a mouse model of HCT116 colorectal cancer. Furthermore, we investigated the therapeutic efficacy of [^225^Ac]Ac-DOTA-TDA-Lipiodol^®^ in a mouse model of CRC to investigate its potential to treat secondary as well as primary liver tumors.

## Methods

### Reagents

All chemicals were purchased from Sigma-Aldrich Chemical Co. (ST. Louis, MO, USA) or Thermo Fisher Scientific (Pittsburgh, PA, USA), unless otherwise specified. DOTA-tris(tert-butyl ester) was purchased from Macrocyclics, Inc. (Dallas, TX, USA). Actinium-225 (Additional file 1: Fig. S1) nitrate was purchased from Oak Ridge National Laboratory (Oak Ridge, TN, USA). Lipiodol^®^ was provided by Guerbet, LLC (Princeton, NJ, USA) through a materials transfer agreement with Dr. Nedrow.

### Radiolabeling

DOTA-TDA was radiolabeled as previously described (Du et al. 2022). Briefly, 15 µL actinium-225 (3.1–7.3 MBq) in 0.2 N HCL was combined with 15 µL 2 M Tris buffer (pH = 7) and 7.5 µL DOTA-TDA in an acid washed Eppendorf tube and heated at 95 °C for 15-min. Post-labeling 2 µL of 1 M gentisic acid was added. For animal studies [^225^Ac]Ac-DOTA-TDA was emulsified in Lipiodol^®^ to provide the αTARE agent, [^225^Ac]Ac-DOTA-TDA-Lipiodol^®^ (see Additional file 1: Table S1).

### Bench-top stability

Post-radiolabeling, the [^225^Ac]Ac-DOTA-TDA in radiolabeling conditions (Tris buffer + gentisic acid) was left on the bench-top at room temperature for 5-days. At 1-h; 1, 3, and 5-days an aliquot of the reaction was assessed by reverse phase HPLC. The HPLC method used a gradient mobile phase of water and acetonitrile containing 0.1% trifluoroacetic acid with 1 mL/min flow rate. The gradient used was 3% acetonitrile raising to 97% over 15-min then held for 4-min until a rapid decrease back to 3% was done over 1-min.

### Serum stability studies

Stability in human serum was assessed for [^225^Ac]Ac-DOTA-TDA. Following radiolabeling, [^225^Ac]Ac-DOTA-TDA (5 μL) was added to 50 μL of human serum, and incubated at 37 °C. At 1-h; 1, 3, and 5-days 50 μL of absolute ethanol (4 °C) was added to an Eppendorf tube containing the serum mixture and placed on ice to precipitate the serum proteins. The Eppendorf tube was than centrifuged at 9000 rpm for 10-min. The supernatant was collected and analyzed by reverse phase HPLC (see Bench-top stability).

### Cell lines

The human colorectal carcinoma cell line HCT116 was purchased from ATCC (Manassas, VA, USA). Cells tested negative for mycoplasma prior to use in mouse model.

### Animal studies

Female and male NCG mice (Strain code: 572) were purchased from Charles River Laboratories (Wilmington, MA, USA). Female and male mice (6–8 weeks-old) were injected subcutaneously with 2 × 10^6^ HCT116 cells in 1:1 sterile PBS:Matrigel, and mice were administered [^225^Ac]Ac-DOTA-TDA-Lipiodol^®^ via an intra-tumoral injection 2–3-weeks post injection (p.i.) of HCT116 cells. Male Sprague–Dawley rats 300-g were purchased from Charles River Laboratories. The healthy non-tumor bearing male rats were administered [^225^Ac]Ac-DOTA-TDA-Lipiodol^®^ via the hepatic artery as described previously (Altomonte et al. 2016). Table 1 summarizes animal studies including numbers and type of experiments.

**Table 1:** Overview of number (n) of animals (female and male NCG mice, and male Sprague–Dawley rats) used in the biodistribution, survival and relative tumor inhibition rate (RTIR) studies

Mice biodistribution studies							
Time-points	35-min	6-h	24-h	72-h	144-h	240-h	504-h
Female mice	n = 3	n = 3	n = 3	n = 3	n = 3	n = 3	n = 3
Male mice	n = 3	n = 3	n = 3	n = 3	n = 3	n = 3	N/A

Rat biodistribution study				
Time-points	1-h	24-h	72-h	144-h
Male rats	n = 3	n = 3	n = 2	n = 2

Compound	[^225^Ac]Ac-DOTA-TDA-Lipiodol^®^	Lipiodol^®^	PBS
*Mice survival studies*			
Female mice	n = 8	n = 8	n = 8
Male mice	n = 6	n = 5	n = 5
*Mice RTIR studies*			
Female mice	n = 5	n = 6	n = 6
Male mice	n = 14	n = 11	n = 9

### Biodistribution studies

Biodistribution studies were performed as previously described (Cortez et al. 2020; Nedrow et al. 2017; Josefsson et al. 2016). In short, HCT116 tumor-bearing female mice with average weight 23.6 ± 1.5 g (range 21.4–26.1 g) and male mice with average weight 28.5 ± 2.4 g (range 24.5–31.5 g) mice (n = 3; per time-point) were injected intratumorally with [^225^Ac]Ac-DOTA-TDA-Lipiodol^®^ (37 kBq, 20 μL) at a 35-min time-interval between the animals in each group and were sacrificed at the following time-points: 35-min; 6, 24, 72, 144, and 240-h p.i. as well as 504-h for the female mice only. Healthy non-tumor bearing male rats with average weight of 354 ± 11 g (range: 340–380 g) were used in the biodistribution study. The male rats (n = 10) were euthanized at the selected time-points 1 (n = 3), 24 (n = 3), 72 (n = 2), and 144-h (n = 2) after administration of 262 ± 29 kBq (range: 206–308 kBq) [^225^Ac]Ac-DOTA-TDA-Lipiodol^®^. Selected organs/tissues were harvested, weighed, and measured in a γ-well counter (PerkinElmer 2480 WIZARD2^®^ Automatic Gamma Counter, MA, USA) (see Additional file 1: M1).

### Therapeutic efficacy

In a colorectal cancer model, male and female mice bearing HCT116 tumors were used to evaluate the therapeutic efficacy of [^225^Ac]Ac-DOTA-TDA-Lipiodol^®^ emulsions. Mice were injected intratumorally with either PBS, Lipiodol^®^ alone, or [^225^Ac]Ac-DOTA-TDA-Lipiodol^®^ (Additional file 1: Table S1). Response to therapy was measured as relative tumor inhibition rate (RTIR) or survival studies. The RTIR was determined by the following expression (Zhou et al. 2019):

1 $$\mathrm{RTIR}=\left[1-\frac{{\left(\frac{{\mathrm{V}}_{\mathrm{Selected Day}}^{\mathrm{Average}}}{{\mathrm{V}}_{0}}\right)}_{\mathrm{Treatment}}}{{\left(\frac{{\mathrm{V}}_{\mathrm{Selected Day}}^{\mathrm{Average}}}{{\mathrm{V}}_{0}}\right)}_{\mathrm{PBS Control}}}\right]\cdot 100\mathrm{\%}, \left[\mathrm{\%}\right].$$

All mice were observed for signs of pain and distress (lethargy, hunched back, etc.), weight loss, and tumor volume (V) calculated using the following expression (Inaba et al. 1989).

2 $$\mathrm{Volume}=0.5\cdot \left(\mathrm{Length}\cdot {\mathrm{Width}}^{2}\right), \left[{\mathrm{mm}}^{3}\right].$$

The mice were euthanized if one of the following conditions were met: (1) Weight loss (> 20%); (2) Primary tumor volume reaching 1500 mm^3^ or 2000 mm^3^ (male RTIR only); (3) Signs of pain or distress (lethargy, hunched back, etc.); (4) Tumor ulceration; or (5) 60-days (Survival studies) or 13/20-days (RTIR only) post-treatment. Survival fractions were plotted as a Kaplan–Meier survival curve using Prism 8 (GraphPad; La Jolla, CA, USA).

### Immunohistochemistry (IHC) studies

Immunohistochemistry (IHC) was performed for the HCT116 tumors in both female and male mice at 24- and 144-h after treatment with [^225^Ac]Ac-DOTA-TDA-Lipiodol^®^. The tumors were embedded in paraffin, sectioned, and stained with hematoxylin and eosin (H&E) or γ-H2AX (see Additional file 1: M2).

### iQID-camera imaging

The iQID-camera system (Miller et al. 2015) was used to image and quantify the activity concentration and distribution of [^225^Ac]Ac-DOTA-TDA-Lipiodol^®^ within the tumor, liver and kidneys at 24- and 144-h after treatment (Additional file 1: M3).

### Dosimetry

The dosimetric calculations were performed according to the Medical Internal Radiation Dose (MIRD) Committee methodology (Sgouros et al. 2010; Loevinger et al. 1991; Bolch et al. 2009), using the measured pharmacokinetic data for [^225^Ac]Ac-DOTA-TDA-Lipiodol^®^ and the free α-particle-emitting daughters francium-221 (including astatine-217) and bismuth-213 (including polonium-213). The dosimetry was performed as previously described (Cortez et al. 2020; Nedrow et al. 2017; Josefsson et al. 2016). The relative biological effectiveness (RBE) for α-particles was incorporated in the dosimetric calculations (Sgouros et al. 2010, 2018b), and the estimated maximum tolerable activity (eMTA) [Bq] was calculated (Additional file 1: M4).

### Statistical analysis

Analysis was performed using the software GraphPad Prism 8 and R version 4.2.3. All data are presented as mean ± SD and a type-I error rate of 0.05 was used for all analyses. Maximum uptake was determined by the mean at each time-point. Retention across organ/tissues were compared with two-way analysis of variance (ANOVA). Specific comparisons between time-points were conducted using analysis of variance (ANOVA), followed by Dunnett's test with 24-h as reference if the ANOVA results were significant. For the sex effect, analysis of covariance (ANCOVA) was used controlling for time when mass is not available. Otherwise, linear regression models were fit with activities against sex, time, and mass. Survival analysis was conducted with the non-parametric Kaplan–Meier methods. Median survival for all treatment groups were estimated and the log rank test was performed to test for a difference between them.

## Results

### Radiolabeling and stability

Radiolabeling yields were ≥ 95%; [^225^Ac]Ac-DOTA-TDA was used without further purification. Bench-top and serum stability studies demonstrated that [^225^Ac]Ac-DOTA-TDA is highly stable with bench-top stability at ≥ 95% radiochemical purity (RCP) over a 3-day period while serum stability was ≥ 90% RCP over 5-days (Additional file 1: Table S2 and Fig. S2).

### Ex vivo biodistribution studies

**Fig. 1:**
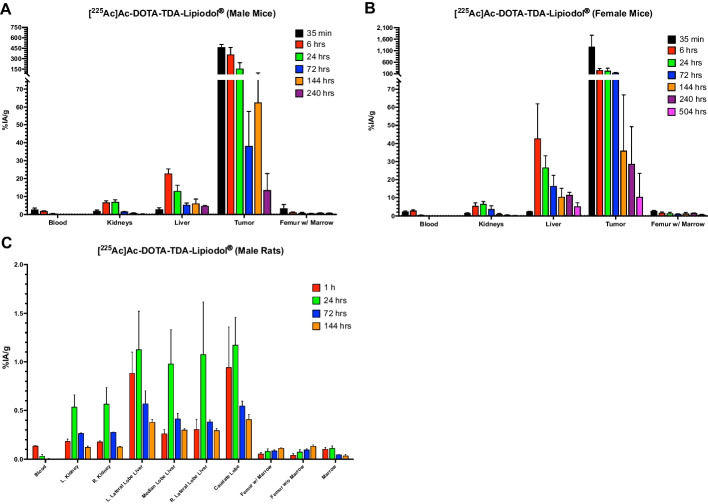
Biodistribution of [^225^Ac]Ac-DOTA-TDA-Lipiodol^®^ of select organs/tissues in: **A** Tumor-bearing male mice at 35-min, 6, 24, 72, 144, and 240-h: **B** Tumor-bearing female mice at 35-min, 6, 24, 72, 144, 240, and 504-h: **C** Non-tumor bearing rats at 1, 24, 72, and 144-h

**Fig. 2:**
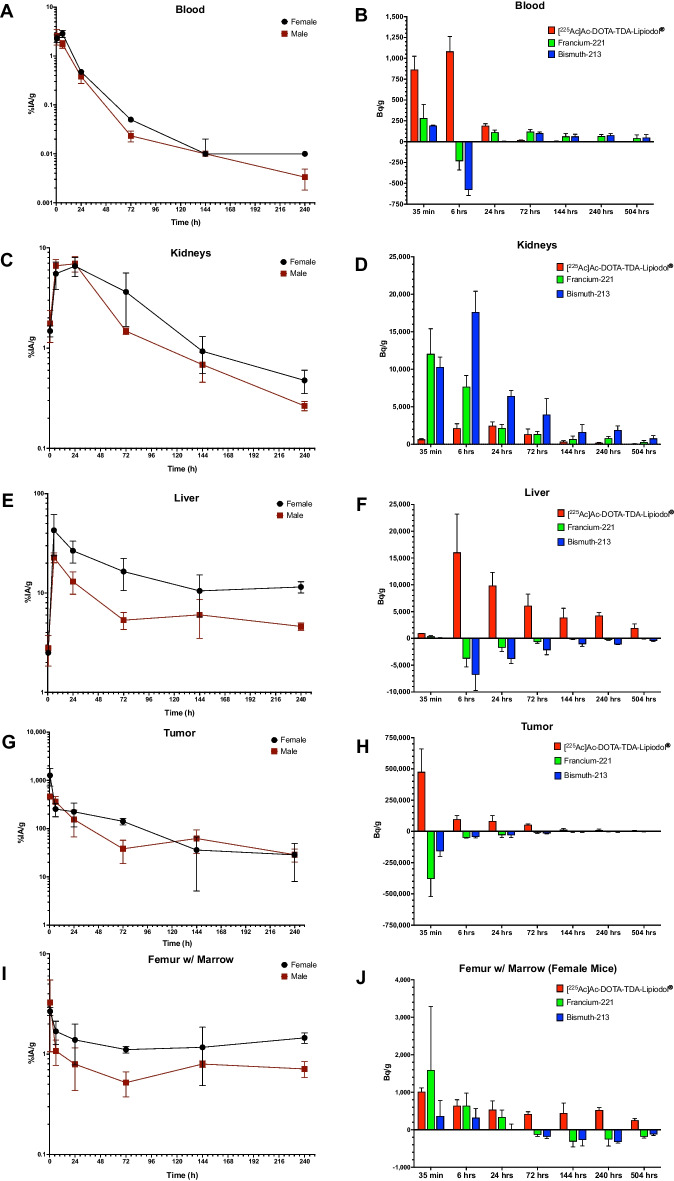
Biodistribution of [^225^Ac]Ac-DOTA-TDA-Lipiodol^®^ in tumor bearing female and male mice in: **A** blood, **C** kidneys, **E** liver, **G** tumor, and **I** femur w/ marrow. Biodistribution of the decay daughters francium-221 (including astatine-217) and bismuth-213 (including polonium-213) in tumor bearing female mice in **B** blood, **D** kidneys, **F** liver, **H** tumor, and **J** femur w/ marrow

Pharmacokinetic data for [^225^Ac]Ac-DOTA-TDA-Lipiodol^®^ in organs/tissues are presented for HCT116 tumor bearing male and female mice (Fig. 1A and B, Additional file 1: Tables S3 and S4, and Fig. S6A and B), and for the non-tumor bearing male rats (Fig. 1C, Additional file 1: Table S5, and Fig. S6C). The pharmacokinetics of the free daughters, francium-221 and bismuth-213, were assessed in the blood, kidneys, liver, tumor, and femur w/ marrow of the female mice (Fig. 2), and in the blood, kidneys, and liver of the male rats (Additional file 1: Fig. S3). In addition, activities at select sites (blood, kidneys, liver, tumor, and femur w/ marrow) were accessed with the analysis of variance (ANOVA) test and followed by Dunnett’s test if the ANOVA result reached statistical significance at an α level of 0.05 with 24-h as the reference; the 24-h timepoint was selected to allow the unretained [^225^Ac]Ac-DOTA-TDA-Lipiodol^®^ to clear following the initial bolus injection as well as allow for sufficient time for the injected free daughters to decay.

#### Blood

##### Mouse model

The blood demonstrated an uptake phase, as expected following an intratumoral injection of [^225^Ac]Ac-DOTA-TDA-Lipiodol^®^, reaching a maximum uptake at 35-min (2.6 ± 0.9%IA/g) for the male and 6-h (2.9 ± 0.5%IA/g) for the female mice (Fig. 2A). The Dunnett’s test indicated significance in activities between 35-min versus 24-h (*p *≤ 0.001), and 6- versus 24-h (*p *≤ 0.01) for both the female and male mice. Figure 2B shows the activity per unit mass (Bq/g) in the blood associated with [^225^Ac]Ac-DOTA-TDA-Lipiodol^®^ and the free daughters in the female mice. We expected the associated activity within the blood at 35-min to include [^225^Ac]Ac-DOTA-TDA-Lipiodol^®^ and its free daughters due to their presence at time of injection as well as their association with the decay of [^225^Ac]Ac-DOTA-TDA-Lipiodol^®^ within the injection site (tumor) and their relocation away from the tumor. The greatest activity (Bq/g) of francium-221 and bismuth-213 was observed at 35-min (192 ± 5 Bq/g and 280 ± 168 Bq/g, respectively). At 6-h, the free daughters associated with the administered dose have mostly decayed and a negative value of activity (Bq/g) is observed for free bismuth-213 and francium-221 at 6-h (-580 ± 67 Bq/g and -233 ± 108 Bq/g, respectively). By 24-h the activity associated with the free daughters, bismuth-213 and francium-221 (0.2 ± 5.4 Bq/g and 111 ± 28 Bq/g, respectively), is again positive indicating that the blood is a supplier and/or transporter of the free relocated daughters.

##### Rat model

Intraarterial delivery of [^225^Ac]Ac-DOTA-TDA-Lipiodol^®^ demonstrated maximum uptake at 1-h (0.13 ± 0.01%IA/g) followed by clearance over a 72-h window (Fig. 1C). The free relocated daughters, francium-221 and bismuth-231, showed a maximum activity in the blood at 1-h after administration (92 ± 53 Bq/g and 40 ± 19 Bq/g, respectively) followed by clearance (Additional file 1: Fig. S3A).

#### Kidneys

##### Mouse model

An uptake phase of [^225^Ac]Ac-DOTA-TDA-Lipiodol^®^ occurs over a 24-h window for both male (35-min: 1.8 ± 0.6, 6-h: 6.6 ± 1.0, 24-h: 6.9 ± 1.2%IA/g, respectively) and female mice (35-min: 1.5 ± 0.2, 6-h: 5.5 ± 1.7, 24-h: 6.6 ± 1.4%IA/g, respectively) in the kidneys, followed by a clearance phase (Fig. 2C). Significant differences in activities between all time-points versus 24-h were observed (*p *≤ 0.05) except for 6- versus 24-h in the female and male mice. However, significance was observed for 6- versus 144-h (*p *≤ 0.001) for the female mice and 6- versus 72-h (*p *≤ 0.001) for the male mice. In the female mice, the kidneys rapidly accumulated both francium-221 and bismuth-213. Figure 2D shows the activity per unit mass (Bq/g) in the kidneys associated with [^225^Ac]Ac-DOTA-TDA-Lipiodol^®^ and the free daughters. Francium-221 reached a maximum uptake at 35-min while bismuth-213 reached a maximum uptake at 6-h, followed by a reduced accumulation of the daughters with time (Fig. 2D). The positive value associated with the free daughters’ activities indicates that they relocate and accumulate in the kidneys.

##### Rat model

The rat model mirrored the findings of the mouse model, with an initial uptake phase over 24-h (0.18 ± 0.02%IA/g [left], 0.18 ± 0.01%IA/g [right]) followed by clearance (Fig. 1C). Francium-221 and bismuth-213 accumulation in the kidneys was highest at 1-h and reduced with time (Additional file 1: Fig. S3B).

#### Liver

##### Mouse model

The normal organ with the highest accumulation of [^225^Ac]Ac-DOTA-TDA-Lipiodol^®^ was the liver. Following the intratumoral injection, the uptake phase occurred over a 6-h window. The maximum uptake was observed at 6-h for the male (23 ± 3%IA/g) and female (43 ± 19%IA/g) mice (Fig. 2E) followed by clearance. Clearance across time-points was not detected, likely due to the supply of [^225^Ac]Ac-DOTA-TDA-Lipiodol^®^ associated with clearance from the tumor. In the female mice, at 35-min the activity associated with the relocated free daughters is minimal and mainly associated with the blood pool in the liver (Fig. 2F). The activity of the free daughters is negative between 6- and 504-h, indicating the free daughters are relocating away from the liver.

##### Rat model

Individual lobes of the liver were measured and demonstrated an uptake phase over a 24-h window followed by a clearance. The caudate liver lobe showed the highest uptake of the lobes at 24-h (1.2 ± 0.3%IA/g) due to its position relative to the hepatic artery. The pharmacokinetics of the free daughters, francium-221 and bismuth-213, were measured in the left lateral liver lobe. Similar to the observation in the mouse model, the activities associated with the free daughters in the liver were negative, indicating that free daughters were relocating away from the liver (Additional file 1: Fig. S3C).

#### Tumor (mouse model only)

##### Mouse model

[^225^Ac]Ac-DOTA-TDA-Lipiodol^®^ (administered intratumorally) had the maximum %IA/g occur at 35-min for both the female and males (1268 ± 505, 465 ± 40%IA/g, respectively), followed by clearance (Fig. 2G). The Dunnett’s analysis indicated significance in activities between 35-min and 24-h (*p *≤ 0.001) for the female and male mice; in addition, the male mice demonstrated a significant change between 6 and 24-h (*p *≤ 0.01) and 24- and 240-h (*p *≤ 0.05). Further analysis demonstrated that the tumor showed a significantly higher [^225^Ac]Ac-DOTA-TDA-Lipiodol^®^ uptake compared to all the other organs/tissues in both the male (*p *< 0.001) and female (*p *< 0.001) mice using a Dunnett’s test. In addition, using a linear regression model and controlling time we show that the retention of [^225^Ac]Ac-DOTA-TDA-Lipiodol^®^ is only significant within the tumor for both females and male mice (*p *≤ 0.001) and no detectable retention was observed for the normal tissues. The distribution of the relocated free daughters, francium-221 and bismuth-213, were assessed in the female mice, the activity (Bq/g) associated with bismuth-213 and francium-221 is negative indicating that the free daughters relocated away from the tumors (Fig. 2H).

#### Femur

##### Mouse model

The femur w/ marrow, similar as the blood, demonstrated an uptake phase of [^225^Ac]Ac-DOTA-TDA-Lipiodol^®^, reaching a maximum uptake at 35-min in female (2.7 ± 0.3%IA/g) and male mice (3.3 ± 2.2% IA/g) (Fig.2I)  followed by clearance. The Dunnett’s test indicates significance in the change between 35-min versus 24-h (*p *≤ 0.001) for the female mice. In the female mice, the free relocated daughters were assessed as shown in Fig. 2J. The early time-points (35-min to 24-h) activity is associated with positive activity indicating the daughters are being supplied to the femur, likely associated with the free daughters present at the time of injection and well as with clearance of unretained [^225^Ac]Ac-DOTA-TDA-Lipiodol^®^’s decay daughters. Following the initial 24-h window, the activities of the daughters are associated with negative activity indicating that the free daughters are relocated away from the femur.

##### Rat model

The accumulation of activity in the marrow was observed at 1-h (0.10 ± 0.02%IA/g), reaching its maximum at 24-h (0.11 ± 0.03%IA/g) followed by clearance to (0.03 ± 0.02%IA/g) at 144-h. Activity accumulation within the femur bone (w/o marrow) increased over the 6-day window, with maximum accumulation at 144-h (0.13 ± 0.01%IA/g), clearance was not observed (Fig. 1C). Accumulation within the femur bone may be associated with loss of actinium-225 from [^225^Ac]Ac-DOTA-TDA-Lipiodol^®^, as free actinium-225 is known to accumulate in bone (Scheinberg and McDevitt 2011). [^225^Ac]Ac-DOTA-TDA demonstrated high stability over a 1-day window, > 95% serum stability, but a slight decrease in stability was observed (> 90% serum stability) at 2–5 days.

#### Male versus female mouse model

Accumulation within the gonads (ovaries or testes) was minimal. Comparison of the male and female mice were performed using analysis of covariance (ANCOVA) within normal tissues and tumors controlled for time. The liver, femur w/ marrow, lungs, pancreas, spleen, and whole-body w/o tumor demonstrated significant differences between the sexes (*p *≤ 0.05). However, when controlled for time and mass using a linear model the liver and whole-body w/o tumor no longer had significance between the sexes. The femur w/ marrow, lungs, pancreas, and spleen still demonstrated differences as well as the tumor (*p *≤ 0.05).

### Therapeutic efficacy

#### Relative tumor inhibition rate (RTIR)

Initial studies in HCT116 tumor-bearing male mice were prematurely ended at thirteen days due to the ulceration of the HCT116 tumors. The RTIR was calculated using Eq. (1) to evaluate the therapeutic efficacy of the [^225^Ac]Ac-DOTA-TDA-Lipiodol^®^ treated male mice. The RTIR studies were repeated in female mice out to 20-days for comparison. For both the male and female mice the RTIR at 11-days (approximately a half-life of actinium-225, T_1/2_ = 9.92-days), were 51.1% and 62.5%, respectively (Additional file 1: Table S6). For the female mice at 20-days (approximately two half-lives of actinium-225) the RTIR decreased to 24.5% due to tumor growth in treated mice; however, it indicates that the [^225^Ac]Ac-DOTA-TDA-Lipiodol^®^ inhibited tumor growth as compared to the controls.

#### Survival studies

**Fig. 3:**
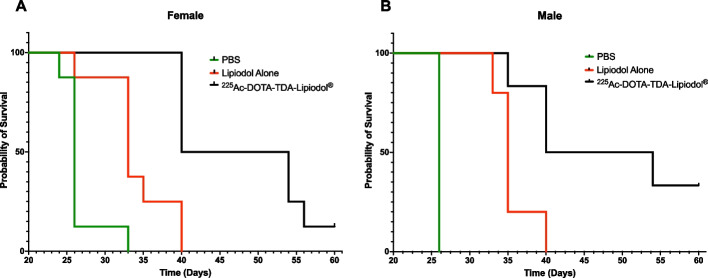
Kaplan–Meier survival plots of tumor-bearing **A** female and **B** male mice receiving the following treatments: (1) PBS (20 μL) (2) Lipiodol^®^ alone (20 μL) or (3) [^225^Ac]Ac-DOTA-TDA-Lipiodol^®^ (37 kBq, 20 μL)

Kaplan–Meier survival curves (Fig. 3) showed significant improvement in survival of HCT116 tumor-bearing male and female mice when treated with [^225^Ac]Ac-DOTA-TDA-Lipiodol^®^ as compared to both the untreated PBS control (*p *≤ 0.01) and Lipiodol^®^ alone (*p *≤ 0.01). The median survival for [^225^Ac]Ac-DOTA-TDA-Lipiodol^®^ treated mice was 47-days for both sexes as compared to controls, 26-days (untreated) and 33–35 days (Lipiodol^®^ alone). In addition, the RTIR for these studies were consistent with the RTIR only studies. Higher RTIRs were observed at 11-days (57.7% male, 75.0% female) then at 20-days (39.5% male, 53.4% females) in the [^225^Ac]Ac-DOTA-TDA-Lipiodol^®^ treated group.

### Immunohistochemistry (IHC) studies

The H&E and γ-H2AX staining of the HCT116 tumors from female and male mice at 24- and 144-h p.i. of [^225^Ac]Ac-DOTA-TDA-Lipiodol^®^ are shown in Additional file 1: Fig. S4. Subcutaneous tumors grew in invasive sheets with areas of central necrosis. Immunostaining for γ-H2AX shows minimal sporadic nuclear staining throughout the solid tumor areas with increased staining in tumor cells around necrotic foci. Similar staining patterns are present in both male and female mice at 24-h and 144-h p.i. of [^225^Ac]Ac-DOTA-TDA-Lipiodol^®^.

### iQID-camera imaging

**Fig. 4:**
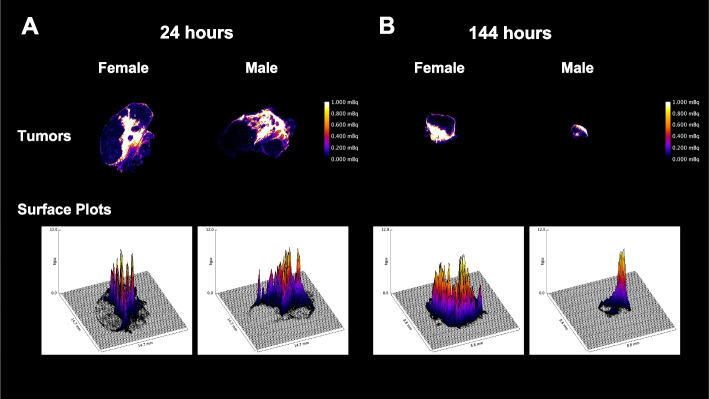
iQID-camera images and surface plots of tumors after administration of [^225^Ac]Ac-DOTA-TDA-Lipiodol^®^ in tumor-bearing female and male mice at **A** 24-h, and **B** 144-h. The scale shows the activity (mBq) present in each sample (The scale was limited to 0–1.0 mBq, higher activities are present in the high activity areas as shown in the bottom row surface plots). The bottom row shows the 3D surface plots for the corresponding iQID-camera image of the tumors with x- and y-axis in length (mm) and the z-axis in units of activity 0–12.0 mBq

iQID-camera images of the activity distribution at 24- and 144-h for [^225^Ac]Ac-DOTA-TDA-Lipiodol^®^ in the liver and kidneys (normal clearance organs) are shown in Additional file 1: Fig. S5. The kidneys show a non-uniform activity distribution with the main uptake occurring within the cortex region for both female and male mice at 24-h: 94% and 97%, and at 144-h: 86% and 85%. The liver shows a relatively uniform distribution of the activity. [^225^Ac]Ac-DOTA-TDA-Lipiodol^®^ shows a non-uniformly distributed within the tumor at 24- and 144-h (Fig. 4).

### Dosimetry

**Fig. 5:**
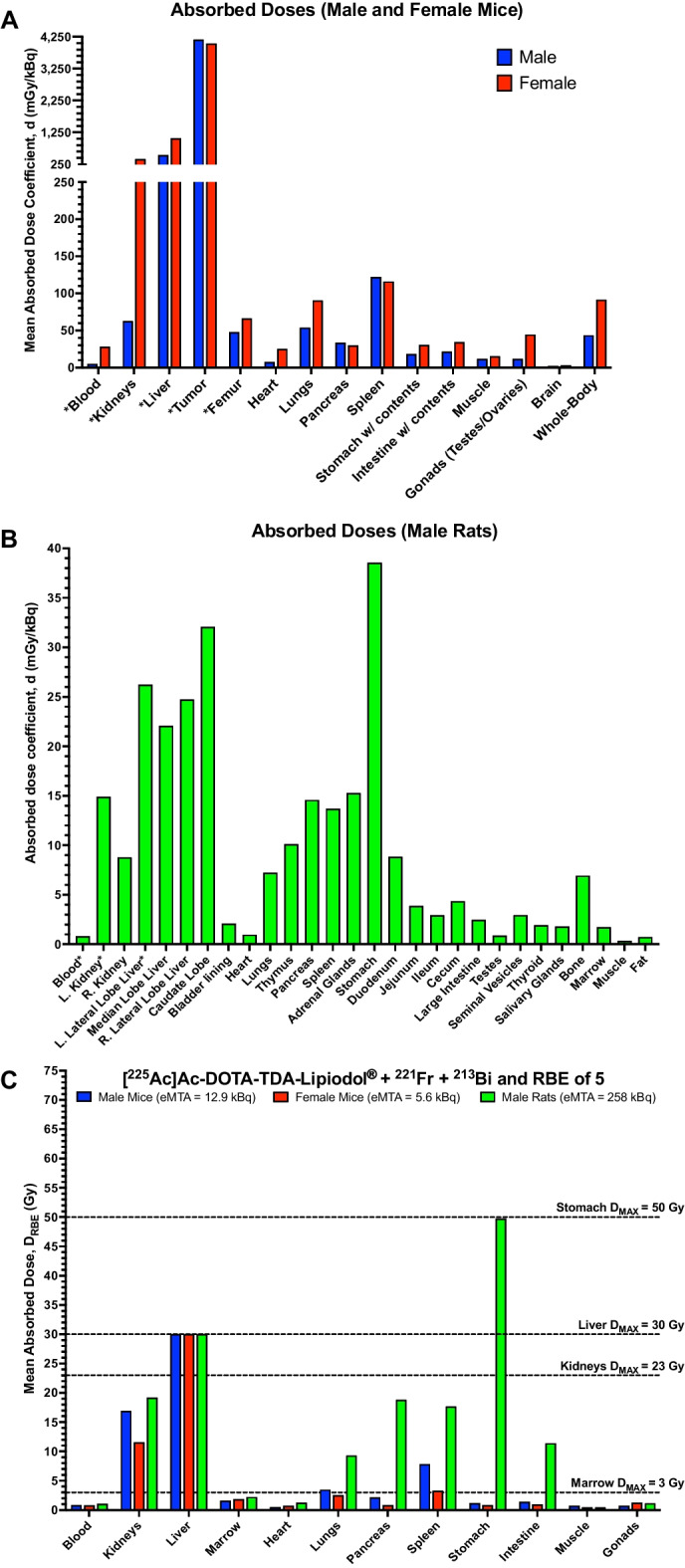
Shows the mean absorbed dose coefficient, d (mGy/kBq), for [^225^Ac]Ac-DOTA-TDA-Lipiodol^®^ in **A** tumor-bearing male (blue bar) and female (red bar) mice, and **B** male non-tumor bearing rats (green bar). **C** The organ/tissue mean absorbed doses using an RBE of 5, D_RBE_ (Gy), and the administered activity based on the calculated estimated maximum tolerable activity (eMTA) in male (blue bar), female (red bar) mice, and male rats (green bar) with liver as the dose limiting organ (maximum tolerable radiation dose (D_MAX_) of 30 Gy). *Absorbed dose coefficients for organs/tissues in female mice and male rats that includes the free daughters

The mean absorbed dose coefficients, $\mathrm{d}$, as well as ${\mathrm{d}}_{\mathrm{RBE}}$, which factors in an RBE-value of 5, for female and male mice, and male rats are shown in Additional file 1: Tables S7–S9, respectively. For the female and male mice, and male rats, the liver was the dose limiting organ and the calculated eMTAs were based on a liver toxicity tolerance of 30 Gy (Emami et al. 1991). In general, a higher mean absorbed dose was observed in the female compared to the male mice, except for the spleen and pancreas that had similar values (Fig. 5A). The mean absorbed dose to the tumor ranged between 23–114 Gy for the female and 47–235 Gy for the male mice, depending on the RBE-value (range 1–5).

For female mice the eMTA ranged between 240 and 1186 Bq/g (whole-body weight), depending on an RBE-value (range 1–5) (Fig. 5C and Additional file 1: Table S7). These calculations take into consideration the relocation of the free in vivo generated α-particle emitting daughters. Table 1 shows that if not considering the relocating daughters the mean absorbed dose was overestimated for the liver and tumor by 14% and 37%, respectively. Regarding the blood and kidneys, the mean absorbed dose would be underestimated by 63% and 76%, respectively.

For male mice the eMTA was based solely on [^225^Ac]Ac-DOTA-TDA-Lipiodol^®^ the eMTA ranged between 396 and 1982 Bq/g (whole-body weight), depending on an RBE-value (range 1–5) (Fig. 5C and Additional file 1: Table S8). The eMTA for the male mice is most likely underestimated as the relocation free daughters was not considered. Assuming the same percent difference as observed for the female mice, the eMTA for the male mice would range between 453 and 2263 Bq/g.

For male rats the eMTA ranged between 728 and 3641 Bq/g (whole-body weight), depending on the RBE-value (range 1–5) (Fig. 5C and Additional file 1: Table S9). The estimates include the relocation of the free daughters measured in the left lateral lobe and the caudate lobe with the highest mean absorbed dose of [^225^Ac]Ac-DOTA-TDA-Lipiodol^®^. If the free daughters were not considered, the mean absorbed dose would be overestimated for the liver by 38%. Regarding the blood and kidneys, the mean absorbed dose would be underestimated by 54% and 45%, respectively (Table 2).

**Table 2:** Mean absorbed dose coefficients, (d) [mGy/kBq], for [^225^Ac]Ac-DOTA-TDA-Lipiodol^®^ and the decay daughters francium-221 (including astatine-217) and bismuth-213 (including polonium-213) in female tumor-bearing mice and non-tumor bearing male Sprague–Dawley rats

Organs/Tissues	[^225^Ac]Ac-DOTA-TDA-Lipiodol^®^ d [mGy/kBq]	Francium-221 d [mGy/kBq]	Bismuth-213 d [mGy/kBq]	Total d [mGy/kBq]
*Female mice*				
Blood	10.3	10.9	6.7	28.0
Kidneys	97.4	121.6	191.2	410
Liver	1213	− 44.5	− 102.8	1066
Tumor	5549	− 962	− 547	4040
*Male rats*				
Blood	0.38	0.25	0.19	0.82
L. kidney	8.3	1.5	5.2	14.9
L. lateral liver lobe	27.7	− 4.0	− 3.6	20.1

Negative numbers mean the daughters relocate away from the organ/tissue, thereby reducing the absorbed dose

Positive numbers mean the daughters relocate and accumulate within this organ/tissue, thereby increasing the absorbed dose

## Discussion

The preliminary investigation of [^225^Ac]Ac-DOTA-TDA-Lipiodol^®^ as an αTARE-agent was conducted in a mouse model of primary liver cancer and in a technical rabbit model (Du et al. 2022), which that [^225^Ac]Ac-DOTA-TDA-Lipiodol^®^ could be selectively delivered to hepatic tumors and induce a significant therapeutic response in a primary model of liver cancer. Herein, we follow up the preliminary investigation of [^225^Ac]Ac-DOTA-TDA-Lipiodol^®^ with a more in-depth look at the pharmacokinetics and dosimetry of the αTARE-agent as well as its relocating daughters. In addition, a CRC murine model was selected to evaluate the potential of [^225^Ac]Ac-DOTA-TDA-Lipiodol^®^ to treat secondary hepatic tumors, as the liver is a common site for metastases.

The development and application of TAT-agents, particularly [^225^Ac]Ac-labeled TAT-agents, has significantly grown over the past decade due to remarkable responses in cancer patients (Sgouros et al. 2020). Previously published studies of [^225^Ac]Ac-labeled TAT-agents have shown them to be highly effective at treating several different types of cancers (Song et al. 2009; Kratochwil et al. 2016; Yadav et al. 2021). In addition, the long physical half-life of actinium-225 (9.92 days) and the low emission of high energy gamma radiation makes handling and transport of [^225^Ac]Ac-labeled TAT-agents favorable to provide highly accessible and potent treatment of cancers (Diamond and Ross 2021; Eckerman and Endo 2008). In this study we demonstrated that [^225^Ac]Ac-DOTA-TDA-Lipiodol^®^ has a favorable stability profile, highlighting the potential of the DOTA-TDA precursor to be amendable to off-site radiolabeling and shipment to facilities for treatment of primary and secondary hepatic tumors (Additional file 1: Fig. S2 and Table S2).

Actinium-225 is a prominent radioisotope in TAT (Eckerman and Endo 2008), with a net total of 4 α- and 2 β-particles emitted between parent and decay daughters (Additional file 1: Fig. S1A). Generally, for [^225^Ac]Ac-labeled TAT-agents the dosage in clinical trials is based on patients’ whole-body weight (Song et al. 2009; Kratochwil et al. 2016; Pelletier et al. 2021). The incorporation of patient specific dosimetry generally improves outcome for patients including for βTARE; clinical trials demonstrated improved response rates and overall survival when patient specific dosimetry was utilized for βTARE (Garin et al. 2021, 2020). The incorporation of patient specific dosimetry for αTARE is desirable. However, dosage based on only the actinium-225 labeled agents, even if patient specific, is still limited as there have been reported toxicities to normal organs, such as the kidneys, associated with the free daughters (Kratochwil et al. 2016; Yadav et al. 2021). To demonstrate the importance of incorporating the daughters’ we evaluated the impact of the daughters on the dose-limiting organ and estimated MTA for [^225^Ac]Ac-DOTA-TDA-Lipiodol^®^ in an non-tumor bearing rat model and in a CRC-tumor bearing mouse model (Figs. 1, 2 and Additional file 1: Fig. S3).

The rat model was selected as a cost-efficient model to assess [^225^Ac]Ac-DOTA-TDA-Lipiodol^®^ pharmacokinetics and the relocation of the free daughters within an in vivo system delivered through the hepatic artery. The mouse model provides a consistent and reproducible model to assess the retention and clearance of the αTARE-agent and its decay daughters within a tumor as well as assess therapeutic efficacy against CRC tumors. However, it should be noted in the mouse subcutaneous model that the intratumoral injections are limited as the dose is mainly deposited along the syringe injection tracts and due to the limited range of α-particles there may be areas of the tumor not exposed to the dose. However, it should be noted that with intraarterial injections we expect [^225^Ac]Ac-DOTA-TDA-Lipiodol^®^ to be better distributed throughout the tumor as we have previously shown that [^225^Ac]Ac-DOTA-TDA-Lipiodol^®^ is distributed more evenly in the tumor when administered intraarterially (VX2-rabbit model) (Du et al. 2022). Unfortunately, the VX2-rabbit model is cost-prohibitive for therapeutic efficacy studies. In both models, we found the liver to have the highest accumulation of [^225^Ac]Ac-DOTA-TDA-Lipiodol^®^ within normal tissues. The higher %IA/g associated with the mouse model is likely due to the supply [^225^Ac]Ac-DOTA-TDA-Lipiodol^®^ clearing from the tumor to the liver followed by clearance through the GI track. The liver was found to be the dose limiting organ with an estimated MTA (eMTA) of 240–1186 Bq/g for female mice (accounting for the daughters relocation), 396–1982 Bq/g for male mice (not accounting for the daughters relocation), and 728–3641 Bq/g (accounting for the daughters relocation) for the male rats (Fig. 5C). The higher eMTA for the rat model is likely due to the lack activity associated with retention and clearance of [^225^Ac]Ac-DOTA-TDA-Lipiodol^®^ within a tumor. It should be noted that the eMTAs are provided as a range based on RBE-values of 1–5, the RBE-value of 5 is a recommendation from the DOE for TAT-agents if there is not additional data to identify an appropriate RBE-value (Feinendegen and McClure 1997). The RBE-value has been reported to vary between 1 and 14 depending on organ/tissue (Sgouros et al. 2018b); however, to our knowledge there is no study that has determined the RBE-value of α-particles for the liver. As the RBE-value is important in TAT due to the high potency of the α-particles and the complexities of their decay chains, future studies to determine the RBE-value within the liver will be performed for this agent.

The free daughters, francium-221 and bismuth-213, were evaluated within the female CRC mouse model and the male rat model. In general, the free daughters relocated away from the tumor and liver, which served as suppliers, and the daughters were transported via the blood where they accumulated in the kidneys’ cortex (See Additional file 1: Fig. S5). It has been reported in the literature that the absorbed doses to the kidneys of [^225^Ac]Ac-labeled TAT-agents’ have approximately 54–69% of the total mean absorbed dose is from free bismuth-213 (including polonium-213) (Cortez et al. 2020; Nedrow et al. 2017; Schwartz et al. 2011; Banerjee et al. 2021). These studies did not consider the contribution from the free daughter francium-221 (including astatine-217), as the longer half-life of bismuth-213 (45.6-min) allows greater relocation to the kidneys within an in vivo system. In this work, we factored in both the francium-221 (including astatine-217) and bismuth-213 (including polonium-213) (Table 2). For the female mice (CRC tumor model), the total mean absorbed dose to the kidneys was 410 mGy/kBq with 23.8% from [^225^Ac]Ac-DOTA-TDA-Lipiodol^®^, 46.6% from free bismuth-213, and 29.6% from free francium-221. The estimated treatment dose based solely on [^225^Ac]Ac-DOTA-TDA-Lipiodol^®^ would underestimate the absorbed dose to the kidneys while overestimating the absorbed dose to the liver, a supplier (loss of free daughters). Similarly, the non-tumor bearing rat model mean absorbed dose to the kidneys was 14.9 mGy/kBq from [^225^Ac]Ac-DOTA-TDA-Lipiodol^®^, 34.7% from free bismuth-213, and 9.8% from free francium-221 (Table 2). The evaluation of [^225^Ac]Ac-DOTA-TDA-Lipiodol^®^ and its free daughters demonstrates that when factoring in the free daughters the estimation of the absorbed dose is more accurate, and highlights the limitation of absorbed dose calculations based only on the [^225^Ac]Ac-labeled TAT-agent or an imaging surrogate alone.

Finally, this work demonstrated therapeutic effectiveness in both male and female mice bearing HCT116 tumors as compared to the controls. The RTIR for female mice were generally greater than RTIR observed in the male mice; however, the median survival for male and female mice were the same, 47-days post-treatment. In the preliminary evaluation of [^225^Ac]Ac-DOTA-TDA-Lipiodol^®^ in the mouse model of liver cancer, the median survival was 45-days, which is consistent with the results presented here. The therapeutic dose administered in these works was based on the preliminary evaluation in the mouse model of liver cancer, with a dose ranging between 1402 and 1968 Bq/g. The doses for the female mice were in the upper range, as their masses were smaller, and the higher Bq/g dose may account for the improved RTIR observed in the female mice as compared to the male mice. The dose 1402–1968 Bq/g fell within the eMTA for male mice and was greater than the eMTA in female mice; however, [^225^Ac]Ac-DOTA-TDA-Lipiodol^®^ treated mice, both male and females, tolerated the dose well as no evidence (weight loss, pain and/or distress) of short-term radiation toxicities were not observed in the mice including ones reaching the 60-day endpoint, suggesting that the dose administered was well-tolerated.

## Conclusion

This study showed that the αTARE-agent [^225^Ac]Ac-DOTA-TDA-Lipiodol^®^ is a promising agent for treatment of hepatic tumors. The therapeutic studies showed a significant increase in survival using [^225^Ac]Ac-DOTA-TDA-Lipiodol^®^ compared with controls for both female and male mice in a mouse model of CRC that is consistent with the therapeutic response observed in a primary model of hepatic tumors. The in-depth evaluation of the [^225^Ac]Ac-DOTA-TDA-Lipiodol^®^ and its daughters, highlights the impact of the daughters on the absorbed doses as well as demonstrating the impact of the shorter-lived decay daughter, francium-221. The stability of [^225^Ac]Ac-DOTA-TDA is promising for centralized manufacturing and distribution, having the potential to provide a highly accessible αTARE-agent for patients with hepatic tumors. In summary, these results further support the continued development of [^225^Ac]Ac-DOTA-TDA-Lipiodol^®^ as a treatment option for treating primary and secondary hepatic tumors.

### Supplementary Information

**Additional file 1.** Supplemental materials.

## Data Availability

The datasets used and/or analyzed during the current study are available from the corresponding author on reasonable request.

## Abbreviations

αTARE: α-Particle-emitting transarterial radioembolization
βTARE: β-Particle-emitting transarterial radioembolization
ANCOVA: Analysis of covariance
ANOVA: Analysis of variance
ATCC: American Type Culture Collection
CRC: Colorectal cancer
DEB-TACE: Drug-eluting bead TACE
DNA: Deoxyribonucleic acid
DOE: Department of Energy
DOTA: Dodecane tetraacetic acid
eMTA: Estimated maximum tolerable activity
H&E: Hematoxylin & eosin
HPLC: High-performance liquid chromatography
IHC: Immunohistochemistry
LET: Linear energy transfer
MIRD: Medical Internal Radiation Dose
PBS: Phosphate buffered saline
p.i.: Post injection
RBE: Relative biological effectiveness
RCP: Radiochemical purity
RTIR: Relative tumor inhibition rate
SM: Supplemental methods
SR: Supplemental results
TACE: Transarterial chemoembolization
TAE: Transarterial embolization
TARE: Transarterial radioembolization
TAT: Targeted alpha therapy
TDA: Tetradecylamine
TRT: Targeted radiotherapy
