# Supplementary material for: Evaluation of the pharmacokinetics, dosimetry, and therapeutic efficacy for the α-particle-emitting transarterial radioembolization (αTARE) agent [225Ac]Ac-DOTA-TDA-Lipiodol® against hepatic tumors
Source: EJNMMI Radiopharm Chem. 2023 Aug 14;8:19. doi: 10.1186/s41181-023-00205-3 (PMC10425307; doi:10.1186/s41181-023-00205-3)
Supplement: Supplementary file 1 — Additional file 1. Supplemental materials. [file 41181_2023_205_MOESM1_ESM.docx]

**Supplemental Materials**

**Evaluation of the pharmacokinetics, dosimetry, and therapeutic efficacy for the α-particle-emitting transarterial radioembolization (αTARE) agent [^225^Ac]Ac-DOTA-TDA-Lipiodol^®^ against hepatic tumors**

*Anders Josefsson, PhD^1^, Angel G. Cortez, BS^1,2^, Harikrishnan Rajkumar, MS^2^, Joseph D. Latoche, BS^1,2^, Ambika P. Jaswal, PhD^3^, Kathryn E. Day, BS^1,2^, Mohammadreza Zarisfi, MD^1^, Lora H. Rigatti, VMD DACVP^2,4^, Ziyu Huang, MS^2^, and Jessie R. Nedrow, PhD^1,2^*

^1^Department of Radiology, University of Pittsburgh School of Medicine, Pittsburgh, PA, USA 
^2^Hillman Cancer Center, University of Pittsburgh School of Medicine, Pittsburgh, PA, USA  
^3^Department of Neurological Surgery, University of Pittsburgh School of Medicine, Pittsburgh, PA, USA 
^4^Division of Laboratory Animal Resources, University of Pittsburgh School of Medicine, Pittsburgh, PA, USA 

**Methods.**

**M1 - Biodistribution studies**

Selected organs/tissues were harvested, weighed, and measured in a γ-well counter (PerkinElmer 2480 WIZARD2^®^ Automatic Gamma Counter, MA, USA) after secular equilibrium had been reached between the parent actinium-225 and its α-particle-emitting decay daughters (francium-221, astatine-217, bismuth-213, and polonium-213 (Supplemental Fig. 1), using the 400–480 keV energy window for bismuth-213 (440.5 keV, yield 26.1%, γ-well counter efficiency 7.1%), for a duration of 10-minutes per sample. The organ/tissues harvested and measured from the mice: blood, kidneys, liver, tumor, femur, heart, lungs, pancreas, spleen, stomach w/ contents, intestines w/ contents, muscles, gonads (ovaries or testes), reminder-of-body. For the male rats: left lateral liver lobe, median liver lobe, right lateral liver lobe, caudate liver lobe, bladder lining, heart, lungs, thymus, spleen, pancreas, kidneys, adrenal glands, stomach, duodenum, jejunum, ileum, cecum, large intestine, testes, seminal vesicles, thyroid, salivary glands, bone w/o marrow, marrow, muscle, reminder-of-body (parts of hide, backbone, muscle, and rib cage). Bone marrow isolation from the femurs were performed using a method developed by Amend *et al.* (1). The percent injected activity per unit mass (%IA/g) was calculated using a pre-determined conversion factor. To measure the free and unbound *in vivo* generated α-particle-emitting decay daughters francium-221 (including astatine-217) and bismuth-213 (including polonium-213) present in the blood, kidneys, liver, tumor, and femur of the mice and blood, left kidney, and left lateral liver lobe in the rats at the time of sacrifice. The samples were harvested as fast as possible after sacrifice and measured in the γ-well counter using the francium-221 and bismuth-213 energy windows simultaneously, 200–240 keV (218.2 keV, yield 11.6%, γ-well counter efficiency 7.0%), and 400–480 keV (440.5 keV, yield 26.1%, γ-well counter efficiency 7.1%), respectively. To measure the free francium-221 (T_1/2_=4.9 minutes) present in each sample they were measured repletely in 30-seconds intervals using the sequential order (blood, kidneys, liver, tumor, and femur) for a total duration of 30–35 minutes. Measuring the photons emitted by francium-221 continuously over time in a sample and fitting the data to a bi-exponential function with half-lives of 9.92 days (actinium-225) and 4.9 minutes (francium-221), respectively. The activity of actinium-225, $A_{\mathrm{Ac}}$ [Bq], and francium-221, $A_{\mathrm{Fr}}$ [Bq], present in the sample at time of sacrifice can be obtained according to the equation:

$A\left( t \right)=\frac{\lambda_{\mathrm{Fr}}}{\lambda_{\mathrm{Fr}}+\lambda_{\mathrm{Ac}}}{\cdot A}_{\mathrm{Ac}}\cdot e^{{-\lambda}_{\mathrm{Ac}}\cdot t}+\left( A_{\mathrm{Fr}}-A_{\mathrm{Ac}} \right)\cdot e^{{-\lambda}_{\mathrm{Fr}}\cdot t}, \left[ \mathrm{Bq} \right].$ (1)

Where $A\left( t \right)$ is the total measured activity [Bq] as a function of time $t$ [s] using the francium-221 energy window, decay rates $\lambda_{\mathrm{Ac}}$ and $\lambda_{\mathrm{Fr}}$ [s^-1^] for actinium-225 and francium-221, respectively. By defining ${A_{\mathrm{fFr}}=(A}_{\mathrm{Fr}}-A_{\mathrm{Ac}})$ as the free francium-221 activity and recognizing that the ratio of decay rates $\left( \lambda_{\mathrm{Fr}}\gg\lambda_{\mathrm{Ac}} \right)$ can be approximated to 1:

$A\left( t \right)\cong A_{\mathrm{Ac}}\cdot e^{{-\lambda}_{\mathrm{Ac}}\cdot t}+A_{\mathrm{fFr}}\cdot e^{{-\lambda}_{\mathrm{Fr}}\cdot t}, \left[ \mathrm{Bq} \right].$ (2)

This approach was used to determine the amount of free francium-221 and labeled actinium-225 in organs and tissues. A similar method was used to determine the amount of free bismuth-213 in each sample previously described (2-4). The activity of actinium-225, francium-221 (including astatine-217) and bismuth-213 (including polonium-213) within the normal tissues were calculated by multiplying the tissue weight with the respective measured activity concentration per unit mass [Bq/g]. The total blood mass, m [g], in a mouse based on its whole-body weight, M [kg], was calculated using the following expression (5):

$m=58.5\cdot M\cdot\rho, \left[ g \right].$ (3A)

Where,$\rho$ [g/cm^3^], is the density of the blood. The total blood volume in a rat was calculated according to the following expression (6):

$BV=0.06\cdot BW+0.77, \left[ \mathrm{ml} \right].$ (3B)

Where $\mathrm{BV}$ is the total blood volume [ml] and $\mathrm{BW}$ the body weight [g] of the rat, this expression is valid for male and female rats weighing between 100–400 gram. The density of the mouse and rat blood was assumed to be the same as in a human 1.06 [g/cm^3^] (7).

Measurements to determine the free daughters present in the organ/tissues at sacrifice were only performed for the HCT116 tumor bearing female mice.

**M2 - Immunohistochemistry (IHC) studies**

The HCT116 tumors from both the male and female mice were immediately harvested and transferred to 10% formalin after sacrifice at 24- and 144-hours p.i. The tumor samples were stored in the formalin for 10 half-lives (∽100-days) of actinium-225 for the radioactivity to decay. The tumor samples were dried and embedded in paraffin using a standard protocol and then cut in 5-μm thick sections for immunohistochemistry (IHC) staining. *Hematoxylin and eosin (H&E) staining:* H&E was performed using SelecTech proprietary reagents from Leica Biosystems (Deer Park, IL, USA). *γ-H2AX staining:* Antigen retrieval was performed using a Citrate buffer (Cell Signaling). The γ-H2AX antibody from Abcam (Cambridge, UK) was applied using a 1:1000 dilution at room temperature. The secondary antibody consisted of a Boost Rabbit HRP Polymer from BioCare Medical (Pacheco, CA, USA). The substrate used was 3,3, Diaminobenzidine + (Cell Signaling), and the slides were counterstained with Hematoxylin.

**M3 - iQID-camera imaging**

The iQID-camera is an *ex vivo* quantitative digital autoradiographical imaging system (QScint Imaging Solutions, AZ, USA), which can image emitted α-particles (8). The female (n=1; per time-point) and male (n=1; per time-point) mice were sacrificed 24- and 144-hours p.i., and the kidneys, liver, and tumor were immediately harvested, embedded in optimal cutting temperature (OCT) and flash-frozen on dry ice (-78.5⁰C). The samples were stored overnight in a -80⁰C freezer to allow the free decay daughters to decay and reach secular equilibrium with [^225^Ac]Ac-DOTA-TDA-Lipiodol^®^. The frozen tissue samples were sectioned using a cryostat TN50 (Tanner Scientific, Sarasota, FL, USA) in 12 μm (kidneys and livers) and 16 μm (tumors) thick sections. The sectioned tissue samples were placed on an scintillator sheet EJ-440 3.2 mg/cm^2^ ZnS:Ag phosphor (Eljen Technologies, Sweetwater ,TX, USA), with an total exposure time of 24-hours in the iQID-camera system. The images were processed and analyzed using the MATLAB^®^ R2022a software (MathWorks Inc., Natick, MA, USA) and ImageJ 1.49b (National Institutes of Health, Bethesda, MD, USA).

**M4 - Dosimetry**

The radiation absorbed doses for [^225^Ac]Ac-DOTA-TDA-Lipiodol^®^ and the free *in vivo* generated α-particle emitting decay daughters were calculated for the blood, kidneys, liver, tumor, femur (mouse), and blood, left kidney, left lateral liver lobe (rat). Regarding the other organs and tissues only the secular equilibrium [^225^Ac]Ac-DOTA-TDA-Lipiodol^®^ radiation absorbed doses were calculated. Mathematical functions were fitted to the time-activity curves (TAC) using the software MATLAB^®^ version R2022a. These mathematical functions were integrated from zero to infinity to calculate the time-integrated activity (TIA), previously called accumulated activity (9). If the data points could not be fitted with a mathematical function, the integral was obtained as the sum of a numerical integration over the measured time-period, and an analytically integrated mono-exponential function based on the last two measure time-points, or as physical decay from the last time-point. The mean absorbed dose, $D$ [Gy], was calculated according to:

$D=\tilde{A}\cdot\frac{\Delta\cdot\varphi}{M}, \left[ \mathrm{Gy} \right].$ (4)

Where $\tilde{A}$ [Bq·s] is the TIA, $M$ [kg] is the weight of the source organ/tissue, $\Delta$ [J/(Bq·s)] the mean energy per nuclear transition, and $\varphi$ [Dimensionless] the absorbed fraction. The mean absorbed dose coefficient, $d$ [Gy/Bq], was calculated according to the following expression:

$d=\frac{D}{A_{0}}, \left[ Gy/Bq \right].$ (5)

Where $A_{0}$[Bq] is the administered activity. Regarding actinium-225 and the decay daughters only the emitted α-particles were considered in the dosimetric calculations, and due to their short range in tissue all the emitted energy was assumed to be deposited locally within each source organ/tissue $\left( \varphi=1 \right)$. Decay data for actinium-225 and its decay daughters were taken from International Commission on Radiological Protection (ICRP) Publication 107 (10). For actinium-225 including the α-particle emitting decay daughters francium-221, astatine-217, bismuth-213, and polonium-213: $\Delta=4.403\cdot{10}^{-12}$ [J/(Bq·s)]; francium-221 and astatine-217: $\Delta=2.142\cdot{10}^{-12}$ [J/(Bq·s)]; bismuth-213 and polonium-213: $\Delta=1.333\cdot{10}^{-12}$ [J/(Bq·s)] (10).

The relative biological effectiveness (RBE) for α-particles has been shown to vary between 1-14 depending on organ/tissue (11). A Department of Energy (DOE) workshop on TAT recommended an RBE-value of 5 pending additional data to identify the most appropriate RBE-value (12). The RBE-value is calculated according to the following expression (13):

$RBE=\frac{D_{\mathrm{REF}}}{D_{\mathrm{TAT}}}, \left[ \mathrm{Dimensionless} \right].$ (6)

Where the biological effect from a reference radiation absorbed dose, $D_{\mathrm{REF}}$ [Gy], compared with the absorbed dose from the TAT-agent, $D_{\mathrm{TAT}}$ [Gy], are the same (11, 13, 14). The absorbed dose that includes the RBE-value, $D_{\mathrm{RBE}}$ [Gy], is calculated according to the following expression (13, 14):

$D_{\mathrm{RBE}}=D\cdot RBE, \left[ \mathrm{Gy} \right].$ (7)

Where $D$ [Gy] is the mean absorbed dose (see Supplementals for details) and the RBE-value is determined for each organ/tissue and radiation type according to equation 4. The corresponding mean absorbed dose coefficient, $d_{\mathrm{RBE}}$ [Gy/Bq], which includes the RBE-value was calculated according to the following expression:

$d_{\mathrm{RBE}}=\frac{D_{\mathrm{RBE}}}{A_{0}}, \left[ Gy/Bq \right].$ (8)

The estimated maximum tolerable activity, $\left( \mathrm{eMTA} \right)$ [Bq], in this study was derived from radiation absorbed dose limits for the TD 5/5 (the probability of 5% complication within 5 years of the treatment) using external radiation therapy (XRT) published by Emami *et al.* (15). Regarding the blood a mean absorbed dose of 4 Gy based on β-particle or Auger electron emitters was shown to be tolerable in nude mice (16, 17). A mean absorbed dose of 3 Gy showed no permanent bone marrow suppression using radiopharmaceutical therapy against differential thyroid cancer (18). The eMTA was calculated according to the following expression:

$eMTA=\frac{D_{\mathrm{MAX}}}{d_{\mathrm{RBE}}}, \left[ \mathrm{Bq} \right].$ (9)

Where $D_{\mathrm{MAX}}$ [Gy] is published tolerated radiation absorbed dose limits, and $d_{\mathrm{RBE}}$ [Gy/Bq] was calculated according to equation 8.

**Tables.**

**Table 1.** Summary of treatments in tumor-bearing male and female mice.

|  | PBS | | | Lipiodol^®^ Alone | | | [^225^Ac]Ac-DOTA-TDA-Lipiodol^®^ | | | |
| --- | --- | --- | --- | --- | --- | --- | --- | --- | --- | --- |
|  | Volume [μL] | n | Average  Tumor  Volume  [mm^3^] | Volume [μL] | n | Average  Tumor  Volume  [mm^3^] | Activity [kBq] | Volume [μL] | n | Average  Tumor Volume  [mm^3^] |
| Male RTIR | 60 | 9 | 472 ± 246 | 60 | 11 | 429 ± 147 | ~55 | 60 | 14 | 685 ± 150 |
| Female RTIR | 20 | 6 | 53.9 ± 29.9 | 20 | 6 | 91.5 ± 59.8 | ~37 | 20 | 5 | 37.7 ± 28.8 |
| Male Survival | 20 | 5 | 59.8 ± 24.1 | 20 | 5 | 46.4 ± 11.7 | ~37 | 20 | 6 | 52.0 ± 41.1 |
| Female Survival | 20 | 8 | 46.4 ± 35.0 | 20 | 8 | 34.5 ± 17.3 | ~37 | 30 | 8 | 39.2 ± 38.4 |

**Table 2.** Assessment of [^225^Ac]Ac-DOTA-TDA stability.

| Time  (h) | Radiochemical purity Bench-Top  (n=3) | Radiochemical purity Serum  (n=3) |
| --- | --- | --- |
| Initial | 99.5 ± 0.23 | 97.9 ± 0.68 |
| 24 | 98.0 ± 1.83 | 96.0 ± 1.34 |
| 48 | 97.0 ± 2.47 | 92.1 ± 2.60 |
| 72 | 95.9 ± 3.01 | 89.9 ± 0.92 |
| 120 | 70.0 ± 18.9 | 92.1 ± 2.39 |

**Table 3.** Biodistribution of [^225^Ac]Ac-DOTA-TDA-Lipiodol^®^ in units of %IA/g and standard deviation (SD) for tumor-bearing female mice, and tumor to blood and muscle ratios.

| Female Mice (%IA/g) | 35 min | 6 hrs | 24 hrs | 72 hrs | 144 hrs | 240 hrs | 504 hrs |
| --- | --- | --- | --- | --- | --- | --- | --- |
| Blood | 2.289 ± 0.412 | 2.867 ± 0.479 | 0.473 ± 0.022 | 0.051 ± 0.005 | 0.009 ± 0.006 | 0.008 ± 0.002 | 0.006 ± 0.005 |
| Kidneys | 1.479 ± 0.186 | 5.509 ± 1.676 | 6.563 ± 1.402 | 3.631 ± 1.983 | 0.932 ± 0.369 | 0.478 ± 0.123 | 0.121 ± 0.085 |
| Liver | 2.503 ± 0.040 | 42.843 ± 19.114 | 26.632 ± 6.629 | 16.456 ± 5.908 | 10.509 ± 4.743 | 11.504 ± 1.495 | 5.225 ± 2.032 |
| Tumor | 1267.98 ± 505.35 | 254.88 ± 78.70 | 223.59 ± 114.70 | 141.88 ± 21.97 | 35.98 ± 30.89 | 28.67 ± 20.66 | 10.45 ± 13.04 |
| Femur | 2.676 ± 0.251 | 1.690 ± 0.438 | 1.383 ± 0.606 | 1.110 ± 0.084 | 1.170 ± 0.688 | 1.454 ± 0.174 | 0.683 ± 0.125 |
| Heart | 1.451 ± 0.194 | 1.788 ± 0.389 | 0.629 ± 0.100 | 0.248 ± 0.002 | 0.219 ± 0.080 | 0.186 ± 0.041 | 0.093 ± 0.068 |
| Lungs | 2.456 ± 0.572 | 4.475 ± 1.475 | 2.730 ± 0.512 | 1.682 ± 0.264 | 0.855 ± 0.198 | 0.765 ± 0.056 | 0.294 ± 0.177 |
| Pancreas | 0.886 ± 0.429 | 1.338 ± 0.379 | 0.843 ± 0.153 | 0.418 ± 0.080 | 0.282 ± 0.112 | 0.269 ± 0.073 | 0.113 ± 0.050 |
| Spleen | 1.986 ± 0.654 | 3.641 ± 0.747 | 3.486 ± 0.251 | 2.059 ± 0.990 | 1.448 ± 0.524 | 1.167 ± 0.536 | 0.372 ± 0.273 |
| Stomach | 2.112 ± 3.105 | 1.321 ± 0.470 | 1.199 ± 1.423 | 0.250 ± 0.057 | 0.087 ± 0.014 | 0.269 ± 0.046 | 0.035 ± 0.007 |
| Intestines | 0.671 ± 0.221 | 6.336 ± 4.074 | 2.686 ± 2.316 | 0.330 ± 0.041 | 0.082 ± 0.021 | 0.072 ± 0.012 | 0.030 ± 0.013 |
| Muscle | 0.929 ± 1.011 | 0.651 ± 0.194 | 0.293 ± 0.089 | 0.144 ± 0.029 | 0.066 ± 0.024 | 0.160 ± 0.105 | 0.041 ± 0.033 |
| Gonads (Ovaries) | 0.764 ± 0.330 | 1.139 ± 0.182 | 0.492 ± 0.254 | 1.207 ± 0.144 | 0.368 ± 0.195 | 0.272 ± 0.057 | 0.457 ± 0.325 |
| Whole-Body | 2.130 ± 1.027 | 4.186 ± 1.373 | 2.538 ± 0.229 | 1.133 ± 0.353 | 0.767 ± 0.284 | 0.780 ± 0.132 | 0.361 ± 0.157 |
| Tumor:Muscle | 1365 | 392 | 763 | 985 | 545 | 179 | 255 |
| Tumor:Blood | 554 | 89 | 473 | 2782 | 3998 | 3584 | 1742 |

**Table 4.** Biodistribution of [^225^Ac]Ac-DOTA-TDA-Lipiodol^®^ in units of %IA/g and standard deviation (SD) for tumor-bearing male mice, and tumor to blood and muscle ratios.

| Male Mice (%IA/g) | 35 min | 6 hrs | 24 hrs | 72 hrs | 144 hrs | 240 hrs |
| --- | --- | --- | --- | --- | --- | --- |
| Blood | 2.589 ± 0.917 | 1.180 ± 0.680 | 0.380 ± 0.107 | 0.021 ± 0.005 | 0.007 ± 0.002 | 0.003 ± 0.002 |
| Kidneys | 1.760 ± 0.619 | 6.664 ± 1.048 | 6.928 ± 1.215 | 1.477 ± 0.116 | 0.683 ± 0.229 | 0.265 ± 0.028 |
| Liver | 2.786 ± 0.945 | 18.852 ± 5.711 | 13.024 ± 3.301 | 5.335 ± 1.035 | 6.036 ± 2.536 | 4.612 ± 0.390 |
| Tumor | 464.52 ± 39.83 | 359.99 ± 101.98 | 154.66 ± 87.26 | 38.19 ± 19.31 | 62.47 ± 31.82 | 13.46 ± 9.36 |
| Femur | 3.270 ± 2.210 | 1.015 ± 0.351 | 0.793 ± 0.360 | 0.522 ± 0.143 | 0.797 ± 0.061 | 0.712 ± 0.129 |
| Heart | 1.549 ± 0.519 | 0.846 ± 0.349 | 0.411 ± 0.097 | 0.129 ± 0.011 | 0.124 ± 0.058 | 0.077 ± 0.016 |
| Lungs | 2.719 ± 0.913 | 2.047 ± 0.466 | 1.309 ± 0.350 | 0.586 ± 0.041 | 0.586 ± 0.239 | 0.388 ± 0.015 |
| Pancreas | 0.702 ± 0.238 | 0.689 ± 0.267 | 0.428 ± 0.094 | 0.188 ± 0.025 | 0.190 ± 0.072 | 0.125 ± 0.005 |
| Spleen | 2.009 ± 0.546 | 1.835 ± 0.417 | 1.383 ± 0.290 | 0.766 ± 0.067 | 0.779 ± 0.197 | 0.578 ± 0.141 |
| Stomach | 4.445 ± 5.801 | 0.820 ± 0.468 | 1.757 ± 0.986 | 0.313 ± 0.014 | 0.066 ± 0.032 | 0.042 ± 0.006 |
| Intestines | 0.597 ± 0.237 | 3.001 ± 0.842 | 2.957 ± 0.470 | 0.271 ± 0.045 | 0.057 ± 0.017 | 0.032 ± 0.002 |
| Muscle | 0.500 ± 0.187 | 0.294 ± 0.089 | 0.159 ± 0.053 | 0.344 ± 0.328 | 0.073 ± 0.045 | 0.160 ± 0.186 |
| Gonads (Testes) | 0.161 ± 0.056 | 0.411 ± 0.061 | 0.294 ± 0.056 | 0.127 ± 0.004 | 0.140 ± 0.043 | 0.104 ± 0.016 |
| Whole-Body | 2.785 ± 0.785 | 2.539 ± 0.563 | 1.705 ± 0.329 | 0.605 ± 0.019 | 0.520 ± 0.174 | 0.350 ± 0.024 |
| Tumor:Muscle | 929 | 1,224 | 973 | 111 | 856 | 84 |
| Tumor:Blood | 179 | 305 | 407 | 1,819 | 8924 | 4486 |

**Table 5.** Biodistribution of [^225^Ac]Ac-DOTA-TDA-Lipiodol^®^ i in units of %IA/g and standard deviation (SD) for non-tumor bearing male rats.

| Male Rat (%IA/g) | 1 h | 24 hrs | 72 hrs | 144 hrs |
| --- | --- | --- | --- | --- |
| Blood | 0.1343 ± 0.0058 | 0.0301 ± 0.0151 | 0.0039 ± 0.0013 | 0.0011 ± 0.0001 |
| L. Kidney | 0.1825 ± 0.0229 | 0.5352 ± 0.1255 | 0.2647 ± 0.0044 | 0.1223 ± 0.0115 |
| R. Kidney | 0.1778 ± 0.0101 | 0.5666 ± 0.1693 | 0.2766 ± 0.0015 | 0.1256 ± 0.0054 |
| L. Lateral Lobe Liver | 0.8835 ± 0.2169 | 1.1260 ± 0.3939 | 0.5703 ± 0.1318 | 0.3802 ± 0.0253 |
| Median Lobe Liver | 0.2621 ± 0.0418 | 0.9792 ± 0.3524 | 0.4141 ± 0.0556 | 0.2988 ± 0.0155 |
| R. Lateral Lobe Liver | 0.3052 ± 0.0995 | 1.0764 ± 0.5402 | 0.3821 ± 0.0219 | 0.2939 ± 0.0226 |
| Caudate Lobe | 0.9439 ± 0.4159 | 1.1722 ± 0.2843 | 0.5462 ± 0.0487 | 0.4092 ± 0.0517 |
| Bladder lining | 0.4107 ± 0.1126 | 0.0846 ± 0.0817 | 0.0399 ± 0.0156 | 0.0244 ± 0.0170 |
| Heart | 0.0887 ± 0.0068 | 0.0359 ± 0.0146 | 0.0137 ± 0.0008 | 0.0095 ± 0.0003 |
| Lungs | 0.2874 ± 0.0932 | 0.1707 ± 0.0662 | 0.0881 ± 0.0073 | 0.0622 ± 0.0158 |
| Thymus | 0.1599 ± 0.1096 | 0.1691 ± 0.0595 | 0.1422 ± 0.0861 | 0.1169 ± 0.0255 |
| Spleen | 0.6223 ± 0.2368 | 0.6111 ± 0.5833 | 0.1725 ± 0.0040 | 0.1365 ± 0.0750 |
| Pancreas | 0.8808 ± 0.1948 | 0.2580 ± 0.1302 | 0.1391 ± 0.0688 | 0.2441 ± 0.2007 |
| Adrenals | 0.5464 ± 0.2505 | 0.1763 ± 0.0218 | 0.1033 ± 0.0358 | 0.0933 ± 0.0027 |
| Stomach | 1.9107 ± 0.1273 | 0.7449 ± 0.3917 | 0.3070 ± 0.1044 | 0.6700 ± 0.3677 |
| Stomach Contents | 0.0420 ± 0.0197 | 0.0343 ± 0.0226 | 0.1301 ± 0.1762 | 0.0125 ± 0.0038 |
| Duodenum | 0.7652 ± 0.3421 | 0.2320 ± 0.0941 | 0.0740 ± 0.0158 | 0.1294 ± 0.1227 |
| Duodenum Contents | 0.1566 ± 0.0682 | 0.4156 ± 0.1498 | 0.0424 ± 0.0245 | 0.0184 ± 0.0068 |
| Jejunum | 0.3757 ± 0.0663 | 0.2020 ± 0.0722 | 0.0684 ± 0.0007 | 0.0396 ± 0.0008 |
| Jejunum Contents | 0.0733 ± 0.0527 | 0.3615 ± 0.1991 | 0.0738 ± 0.0586 | 0.0173 ± 0.0025 |
| Ileum | 0.3630 ± 0.0506 | 0.2129 ± 0.1064 | 0.0466 ± 0.0021 | 0.0435 ± 0.0191 |
| Ileum Contents | 0.0323 ± 0.0184 | 0.7068 ± 0.4914 | 0.1477 ± 0.1257 | 0.0298 ± 0.0029 |
| Cecum | 0.4553 ± 0.2785 | 0.2604 ± 0.0805 | 0.0796 ± 0.0242 | 0.0740 ± 0.0408 |
| Cecum Contents | 0.0127 ± 0.0092 | 1.2775 ± 0.1061 | 0.1082 ± 0.0473 | 0.0204 ± 0.0032 |
| Large Intestine | 0.3497 ± 0.0675 | 0.1469 ± 0.0300 | 0.0484 ± 0.0100 | 0.0244 ± 0.0002 |
| Large Intestine Contents | 0.0138 ± 0.0041 | 2.1238 ± 0.3703 | 0.1729 ± 0.0724 | 0.0234 ± 0.0006 |
| Gonads (Testes) | 0.0093 ± 0.0038 | 0.0457 ± 0.0281 | 0.0476 ± 0.0262 | 0.0136 ± 0.0020 |
| Seminal Vesicles | 0.1113 ± 0.0140 | 0.1591 ± 0.0974 | 0.0952 ± 0.0329 | 0.0447 ± 0.0119 |
| Thyroid | 0.0882 ± 0.0380 | 0.0795 ± 0.0252 | 0.0408 ± 0.0151 | 0.0340 ± 0.0105 |
| Salivary Glands | 0.0746 ± 0.0136 | 0.0874 ± 0.0296 | 0.0528 ± 0.0042 | 0.0292 ± 0.0101 |
| Bone w/o Marrow | 0.0411 ± 0.0154 | 0.0729 ± 0.0259 | 0.0960 ± 0.0106 | 0.1335 ± 0.0117 |
| Bone w/ Marrow | 0.0535 ± 0.0133 | 0.0801 ± 0.0256 | 0.0856 ± 0.0095 | 0.1125 ± 0.0040 |
| Marrow | 0.1031 ± 0.0189 | 0.1104 ± 0.0258 | 0.0453 ± 0.0006 | 0.0329 ± 0.0157 |
| Muscle | 0.0561 ± 0.0250 | 0.0134 ± 0.0039 | 0.0056 ± 0.0000 | 0.0037 ± 0.0009 |
| Fat | 0.2144 ± 0.0656 | 0.0429 ± 0.0489 | 0.0193 ± 0.0037 | 0.0059 ± 0.0017 |
| Reminder-Of-Body | 0.1201 ± 0.0272 | 0.0290 ± 0.0186 | 0.0218 ± 0.0057 | 0.0151 ± 0.0015 |

**Table 6.** RTIR in HCT116 tumor-bearing male and female NCG mice treated with [^225^Ac]Ac-DOTA-TDA-Lipiodol^®^.

|  | 11 days | 13 days | 20 days |
| --- | --- | --- | --- |
| Male Mice | 51.1% | 51.8% | N/A |
| Female Mice | 62.5% | 54.6% | 24.5% |

**Table 7.** The mean absorbed dose coefficients (d) [mGy/kBq], mean absorbed dose coefficients including an RBE of 5 (d_RBE_) [mGy/kBq], and the estimated maximum tolerable activity (eMTA) [kBq], for organs/tissues in tumor bearing female mice for [^225^Ac]Ac-DOTA-TDA-Lipiodol^®^. The eMTA was based on the calculated d_RBE_ and published maximum tolerable absorbed dose values (D_MAX_) [Gy].

| Organs/Tissues | d  [mGy/kBq] | d_RBE_  [mGy/kBq] | D_MAX_  [Gy] | eMTA  [kBq] |
| --- | --- | --- | --- | --- |
| Blood | 28.0^†^ | 140^†^ | 4.0^‡^ | 28.6 |
| Kidneys | 410^†^ | 2,050^†^ | 23.0^§^ | 11.2 |
| **Liver** | **1,066^†^** | **5,328^†^** | **30.0^§^** | **5.6** |
| Femur (Bone + Marrow) | 66.2^†^ | 331^†^ | 3.0^*^ | 9.1 |
| Tumor | 4,040^†^ | 20,200^†^ | - | - |
| Heart | 25.1 | 126 | 45.0^§^ | 358 |
| Lungs | 90.5 | 452 | 17.5^§^ | 38.7 |
| Pancreas | 30.0 | 150 | - | - |
| Spleen | 116 | 582 | - | - |
| Stomach w/ Contents | 30.3 | 152 | 50.0^§^ | 930 |
| Intestine w/ Contents | 34.3 | 172 | 40.0^§^ | 233 |
| Muscle | 15.1 | 75.4 | - | - |
| Ovaries | 44.5 | 223 | - | - |
| Whole-Body w/o Tumor | 91.4 | 457 | 3.0^*^ | 6.6 |

^†^Including [^225^Ac]Ac-DOTA-TDA-Lipiodol^®^, and the contribution from the free and unbound daughters francium-221, astatine-217, bismuth-213, and polonium-213.

^‡^A mean absorbed dose of 4 Gy based on β-particle or Auger electron emitters was shown to be tolerable in nude mice (16, 17).

^§^The estimated maximum tolerable radiation absorbed dose limits for the TD 5/5 (the probability of 5% complication within 5 years of the treatment) using external radiation therapy (XRT) published by Emami et al. (15).

^*^A mean absorbed dose of 3 Gy showed no permanent bone marrow suppression using radiopharmaceutical therapy (RPT) against differential thyroid cancer (18).

**Table 8.** The mean absorbed dose coefficients (d) [mGy/kBq], mean absorbed dose coefficients including an RBE of 5 (d_RBE_) [mGy/kBq], and the estimated maximum tolerable activity (eMTA) [kBq], for organs/tissues in tumor bearing male mice for [^225^Ac]Ac-DOTA-TDA-Lipiodol^®^. The eMTA was based on the calculated d_RBE_ and published maximum tolerable absorbed dose values (D_MAX_) [Gy].

| Organs/Tissues | d  [mGy/kBq] | d_RBE_  [mGy/kBq] | D_MAX_  [Gy] | eMTA  [kBq] |
| --- | --- | --- | --- | --- |
| Blood | 4.7 | 23.3 | 4.0^‡^ | 171 |
| Kidneys | 62.6 | 313 | 23.0^§^ | 73.5 |
| **Liver** | **532** | **2,660** | **30.0^§^** | **11.3** |
| Femur (Bone + Marrow) | 47.8 | 239 | 3.0^*^ | 12.5 |
| Tumor | 4,160 | 20,797 | - | - |
| Heart | 7.4 | 37.0 | 45.0^§^ | 1,200 |
| Lungs | 53.9 | 270 | 17.5^§^ | 64.9 |
| Pancreas | 33.6 | 168 | - | - |
| Spleen | 122 | 610 | - | - |
| Stomach | 18.4 | 92.1 | 50.0^§^ | 540 |
| Intestine | 21.6 | 108 | 40.0^§^ | 370 |
| Muscle | 11.4 | 57.1 | - | - |
| Testes | 11.4 | 57.1 | - | - |
| Whole-Body w/o Tumor | 43.6 | 218 | 3.0^*^ | 13.8 |

^‡^A mean absorbed dose of 4 Gy based on β-particle or Auger electron emitters was shown to be tolerable in nude mice (16, 17).

^§^The estimated maximum tolerable radiation absorbed dose limits for the TD 5/5 (the probability of 5% complication within 5 years of the treatment) using external radiation therapy (XRT) published by Emami et al. (15).

^*^A mean absorbed dose of 3 Gy showed no permanent bone marrow suppression using radiopharmaceutical therapy (RPT) against differential thyroid cancer (18).

**Table 9.** The mean absorbed dose coefficients (d) [mGy/kBq], mean absorbed dose coefficients including an RBE of 5 (d_RBE_) [mGy/kBq], and the estimated maximum tolerable activity (eMTA) [kBq], for organs/tissues in non-tumor bearing male rats for [^225^Ac]Ac-DOTA-TDA-Lipiodol^®^. The eMTA was based on the calculated d_RBE_ and published maximum tolerable absorbed dose values (D_MAX_) [Gy].

| Organs/Tissues | d  [mGy/kBq] | d_RBE_  [mGy/kBq] | D_MAX_  [Gy] | eMTA  [kBq] |
| --- | --- | --- | --- | --- |
| Blood | 0.82^†^ | 4.0^†^ | 4.0^‡^ | 979 |
| L. Kidney | 14.9^†^ | 74.4^†^ | 23.0^§^ | 430 |
| R. Kidney | 8.8 | 43.9 | 23.0^§^ | 524 |
| L. Lateral Liver Lobe | 20.1^†^ | 100.4^†^ | 30.0^§^ | 299 |
| Median Liver Lobe | 22.1 | 110.4 | 30.0^§^ | 272 |
| R. Lateral Liver Lobe | 24.7 | 123.7 | 30.0^§^ | 243 |
| **Caudate Liver Lobe** | **32.1** | **160.3** | **30.0^§^** | **187** |
| Heart | 0.96 | 4.8 | 45.0^§^ | 9,346 |
| Lungs | 7.2 | 36.1 | 17.5^§^ | 485 |
| Pancreas | 14.6 | 72.9 | - | - |
| Spleen | 13.7 | 68.5 | - | - |
| Salivary Glands | 1.80 | 9.0 | 30.0^§^ | 3,335 |
| Stomach | 38.6 | 192.8 | 50.0^§^ | 259 |
| Small Intestine (Duodenum) | 8.8 | 44.2 | 40.0^§^ | 905 |
| Large Intestine (Colon) | 2.5 | 12.3 | 45.0^§^ | 3,662 |
| Muscle | 0.34 | 1.7 | - | - |
| Testes | 0.89 | 4.4 | - | - |
| Bone | 6.9 | 34.7 | 52.0^§^ | 1,498 |
| Marrow | 1.7 | 8.6 | 3.0^*^ | 347 |

^†^Including [^225^Ac]Ac-DOTA-TDA-Lipiodol^®^, and the contribution from the free and unbound daughters francium-221, astatine-217, bismuth-213, and polonium-213.

^‡^A mean absorbed dose of 4 Gy based on β-particle or Auger electron emitters was shown to be tolerable in nude mice (16, 17).

^§^The estimated maximum tolerable radiation absorbed dose limits for the TD 5/5 (the probability of 5% complication within 5 years of the treatment) using external radiation therapy (XRT) published by Emami et al. (15).

^*^A mean absorbed dose of 3 Gy showed no permanent bone marrow suppression using radiopharmaceutical therapy (RPT) against differential thyroid cancer (18).

**Figures.**

**Fig. 1. A)** Decay scheme of actinium-225 and its decay daughters. **B)** The decay daughters activity (kBq) as a function of time to reach secular equilibrium with the parent actinium-225 (excluding thallium-209 due to low yield and beta-particle emitter).

**Fig. 2.** Representative HPLCs of **A)** Bench-Top Stability and **B)** Serum Stability of [^225^Ac]Ac-DOTA-TDA.

**Fig. 3.** Biodistribution of [^225^Ac]Ac-DOTA-TDA-Lipiodol^®^ and the decay daughters francium-221 (including astatine-217) and bismuth-213 (including polonium-213) in non-tumor bearing male Sprague-Dawley rats for the organs and tissues **A)** Blood, **B)** Kidneys, and **C)** Liver.

**Fig. 4.** Shows H&E and γ-H2AX staining of the HCT116 tumors from female (top two rows) and male (bottom two rows) mice at 24- and 144-hours p.i. of [^225^Ac]Ac-DOTA-TDA-Lipiodol^®^.

**Fig. 5.** iQID-camera images of the liver and kidneys of ~37 kBq [^225^Ac]Ac-DOTA-TDA-Lipiodol^®^ p.i. injection in HCT116 tumor bearing female and male NCG mice at A) 24-hours, and B) 144-hours. The tissue slices are 12 μm thick sections and were imaged for a total duration of 24-hours (exposure time). The calibration bar shows the activity (mBq) present in each sample. Note different scale for kidneys at 144-hours p.i.

**
Fig. 6.** Biodistribution [^225^Ac]Ac-DOTA-TDA-Lipiodol^®^ in **A)** HCT116 tumor-bearing male mice at 35 min, 6-, 24-, 72-, 144-, and 240-hours **B)** HCT116 tumor-bearing female mice at 35 min, 6-, 24-, 72-, 144-, 240-, and 504-hours: **C)** Non-tumor bearing male rats at 1, 24, 72, and 144 hours.

**Supplemental References**

1. Amend SR, Valkenburg KC, Pienta KJ. Murine Hind Limb Long Bone Dissection and Bone Marrow Isolation. J Vis Exp. 2016(110).

2. Cortez A, Josefsson A, McCarty G, Shtekler AE, Rao A, Austin Z, et al. Evaluation of [(225)Ac]Ac-DOTA-anti-VLA-4 for targeted alpha therapy of metastatic melanoma. Nucl Med Biol. 2020;88-89:62-72.

3. Nedrow JR, Josefsson A, Park S, Back T, Hobbs RF, Brayton C, et al. Pharmacokinetics, microscale distribution, and dosimetry of alpha-emitter-labeled anti-PD-L1 antibodies in an immune competent transgenic breast cancer model. EJNMMI Res. 2017;7(1):57.

4. Banerjee SR, Lisok A, Minn I, Josefsson A, Kumar V, Brummet M, et al. Preclinical Evaluation of (213)Bi- and (225)Ac-Labeled Low-Molecular-Weight Compounds for Radiopharmaceutical Therapy of Prostate Cancer. J Nucl Med. 2021;62(7):980-8.

5. National Center for the Replacement Refinement & Reduction of Animals in Research (NC3RS) hwnrou. <https://www.nc3rs.org.uk/>, 2018.

6. Lee HB, Blaufox MD. Blood volume in the rat. J Nucl Med. 1985;26(1):72-6.

7. ICRP. ICRP publication 110. Realistic reference phantoms: an ICRP/ICRU joint effort. A report of adult reference computational phantoms. Ann ICRP. 2009;39(2):1-164.

8. Miller BW, Frost SH, Frayo SL, Kenoyer AL, Santos E, Jones JC, et al. Quantitative single-particle digital autoradiography with alpha-particle emitters for targeted radionuclide therapy using the iQID camera. Med Phys. 2015;42(7):4094-105.

9. Bolch WE, Eckerman KF, Sgouros G, Thomas SR. MIRD pamphlet No. 21: a generalized schema for radiopharmaceutical dosimetry--standardization of nomenclature. J Nucl Med. 2009;50(3):477-84.

10. Eckerman K, Endo A. ICRP Publication 107. Nuclear decay data for dosimetric calculations. Ann ICRP. 2008;38(3):7-96.

11. Sgouros G, Allen, B.J., Brill, A.B., Fisher, D.R., Hobbs, R.F., Howell, R.W., McDevitt, M.R., Meredith, R.F., Miller, B.W., Palm, S. Roeske, J.C., Sofou, S., Song, H., Torgue, J., Zalutsky, M.R. MIRD Radiobiology and Dosimtery for Radiopharmaceutical Therapy with Alpha-Particle Emitters. Sgouros G, editor. Reston, VA2015.

12. Feinendegen LE, McClure JJ. Meeting report - Alpha-emitters for medical therapy - Workshop of the United States Department of Energy - Denver, Colorado, May 30-31, 1996. Radiat Res. 1997;148(2):195-201.

13. Sgouros G, Roeske JC, McDevitt MR, Palm S, Allen BJ, Fisher DR, et al. MIRD Pamphlet No. 22 (abridged): radiobiology and dosimetry of alpha-particle emitters for targeted radionuclide therapy. J Nucl Med. 2010;51(2):311-28.

14. Sgouros G, Hobbs R, Josefsson A. Dosimetry and Radiobiology of Alpha-Particle Emitting Radionuclides. Curr Radiopharm. 2018;11(3):209-14.

15. Emami B, Lyman J, Brown A, Coia L, Goitein M, Munzenrider JE, et al. Tolerance of normal tissue to therapeutic irradiation. Int J Radiat Oncol Biol Phys. 1991;21(1):109-22.

16. Behr TM, Behe M, Sgouros G. Correlation of red marrow radiation dosimetry with myelotoxicity: empirical factors influencing the radiation-induced myelotoxicity of radiolabeled antibodies, fragments and peptides in pre-clinical and clinical settings. Cancer Biother Radiopharm. 2002;17(4):445-64.

17. Behr TM, Sgouros G, Stabin MG, Behe M, Angerstein C, Blumenthal RD, et al. Studies on the red marrow dosimetry in radioimmunotherapy: an experimental investigation of factors influencing the radiation-induced myelotoxicity in therapy with beta-, Auger/conversion electron-, or alpha-emitters. Clin Cancer Res. 1999;5(10 Suppl):3031s-43s.

18. Dorn R, Kopp J, Vogt H, Heidenreich P, Carroll RG, Gulec SA. Dosimetry-guided radioactive iodine treatment in patients with metastatic differentiated thyroid cancer: largest safe dose using a risk-adapted approach. J Nucl Med. 2003;44(3):451-6.
